# Dynamic Hand Gesture Recognition in In-Vehicle Environment Based on FMCW Radar and Transformer

**DOI:** 10.3390/s21196368

**Published:** 2021-09-24

**Authors:** Lianqing Zheng, Jie Bai, Xichan Zhu, Libo Huang, Chewu Shan, Qiong Wu, Lei Zhang

**Affiliations:** 1 Institute of Intelligent Vehicles, School of Automotive Studies, Tongji University, Shanghai 201804, China; zhenglianqing@tongji.edu.cn (L.Z.); zhuxichan@tongji.edu.cn (X.Z.); 1911085@tongji.edu.cn (C.S.); 2 School of Information and Electricity, Zhejiang University City College, Hangzhou 310015, China; baij@zucc.edu.cn; 3 Technical Center of Anhui Jianghuai Automobile Co., Ltd., Hefei 230601, China; qiong.wu@jac.com.cn (Q.W.); zhlei@jac.com.cn (L.Z.)

**Keywords:** gesture recognition, human-computer interaction, FMCW radar, deep learning, transformer

## Abstract

Hand gesture recognition technology plays an important role in human-computer interaction and in-vehicle entertainment. Under in-vehicle conditions, it is a great challenge to design gesture recognition systems due to variable driving conditions, complex backgrounds, and diversified gestures. In this paper, we propose a gesture recognition system based on frequency-modulated continuous-wave (FMCW) radar and transformer for an in-vehicle environment. Firstly, the original range-Doppler maps (RDMs), range-azimuth maps (RAMs), and range-elevation maps (REMs) of the time sequence of each gesture are obtained by radar signal processing. Then we preprocess the obtained data frames by region of interest (ROI) extraction, vibration removal algorithm, background removal algorithm, and standardization. We propose a transformer-based radar gesture recognition network named RGTNet. It fully extracts and fuses the spatial-temporal information of radar feature maps to complete the classification of various gestures. The experimental results show that our method can better complete the eight gesture classification tasks in the in-vehicle environment. The recognition accuracy is 97.56%.

## 1. Introduction

In recent years, with the development of electronic technology and artificial intelligence [1], human–computer interaction technology has received much attention. Among them, gesture recognition is one of the most important branches in the field of human-computer interaction [2]. It has been widely used in industrial production [3,4], including smart homes, virtual reality, and intelligent cockpits.

According to the source of gesture signals, gesture recognition is mainly classified into visual-based signals, biological-based signals, inertial sensor-based signals and radar-based signals. The first is gesture recognition methods based on visual signals. In [5], the authors proposed a part-based gesture recognition system with some robustness using Kinect as a sensor. Using the new metric, namely Finger-Earth Mover’s Distance, the accuracy rate of gesture recognition reached more than 90%. In [6], the authors collect continuous multi-frame images of dynamic gestures and use three-dimension separable convolution for gesture recognition. Dhingra et al. [7] applied the attention mechanism in the three-dimension convolutional neural networks (3DCNN) model and learned features at different scales to obtain good classification results. Coelho et al. [8] used convolutional neural networks to extract and classify images through the acquired RGB and depth maps to accomplish gesture recognition.

The next is gesture recognition methods based on biological signals. It is mainly used to capture original signals through human wearable devices, mainly around electromyography (EMG) [9]. Chen et al. [10] selected 30 designed muscle activation patterns of finger joints, elbow joints and wrist joints to train a classifier to complete gesture recognition by extracting the relevant features from the collected EMG. Matsubara et al. [11] proposed a bilinear model of EMG signals consisting of user dependence and motion dependence. After detecting the EMG signal, the model decomposes it into user dependence and motion dependence to achieve classification and recognition of different gestures from multiple users with an accuracy of 73%. Lu et al. [12] used a combination of EMG and accelerometer to identify the 19 gestures that were designed by capturing the difference between the two signals during different hand movements. Zhang et al. [13] obtained gesture information from accelerometers and multi-channel EMG sensors attached to the human hand and then combined the Markov model and decision tree model to obtain the results.

In terms of inertial sensor-based signals, the Samsung Research Institute [14] proposed a gesture recognition system using accelerometers and gyroscopes. It obtained the trajectory of the hand in the two-dimensional plane by the angular velocity and acceleration of the user’s hand in three-dimensional space during its movement. Subsequently, it used Bayesian networks to model and match the gestures. Danial et al. [15] designed a smartwatch for text input through gestures. The features corresponding to the gestures of 26 English letters were extracted by the accelerometer added inside the watch. The recognition accuracy was only 71% since some of the letters were extracted with similar features, and the overall number of letters was large.

This paper considers gesture recognition in the in-vehicle environment, which belongs to the field of in-vehicle entertainment and intelligent cockpit. The gesture recognition application under an in-vehicle environment can improve driving safety and prevent the driver from being distracted by operating the vehicle screen. It can also increase the fun of driving and the intelligence of the vehicle. For example, through the effective recognition of gestures, users can switch songs, adjust the volume, control the vehicle windows and other functions.

Gesture recognition based on biological signals and inertial sensor signals mainly adopts wearable devices and multiple sensors, which is unsuitable for practicability and convenience. The accuracy of the gesture recognition system based on visual signals, such as Volkswagen Golf, is reduced due to poor lighting and illumination in the vehicle. On the other hand, radar has low requirements for light and intense penetration and can protect users’ privacy, making it more suitable for use in the vehicle and different environments. [16]. Therefore, gesture recognition schemes based on radar signals have begun to emerge in recent years. Gao et al. [17] used 2.4 GHz Doppler radar as a sensor to extract the zero-crossing features of radar baseband signals corresponding to different gestures and then perform subsequent processing to achieve gesture classification. In [18], the 24 GHz millimetre-wave (MMW) radar was used to recognize the three gestures. The predictive classification was achieved by training a convolutional neural network. Kim et al. [19] collected data of eight gestures by Doppler radar. They obtained Doppler spectrograms by short-time Fourier transform to extract features. Finally, They fed them into a convolutional neural network (CNN) for training and prediction. Smith et al. [20] developed an in-vehicle entertainment gesture recognition system based on Google soli radar [21]. The system used a random forest classifier to recognize six preset gestures. Doppler radar can only obtain velocity information of the measured object and cannot obtain specific position-distance information [22]. Therefore, FMCW radar is widely used, which can return radial velocity, range and angle information of the measured target and thus have more characteristic representations for different gestures. In [23], a two-dimensional fast Fourier transform (FFT) was used to generate a range-Doppler map (RDM) to extract gesture features. However, it does not consider the angle information, resulting in poor gesture recognition for horizontal and vertical movements. The literature [22,24] used CNN and Long Short-Term Memory (LSTM) to learn gesture features on time sequences using temporal RDMs and RAMs as inputs. They used the continuous frame information and the extracted time-domain information, respectively. Zhang et al. [25] proposed an MMW radar gesture recognition system named latern, which was trained end-to-end by 3DCNN combined with LSTM. It also performed different dynamic gesture recognition by Connectionist Temporal Classification (CTC) algorithm with an accuracy of 96%. In [26], the authors generated features for gesture classification from range-Doppler maps of FMCW radar. A wrapper-based feature selection algorithm combined with a quantum-inspired evolutionary algorithm (QEA) was used to extract highly relevant features. The algorithm improves the gesture recognition accuracy of the radar system. In [27], the authors used principal component analysis (PCA) and t-distributed stochastic neighbour embedding (t-SNE) algorithm, combined with machine learning techniques, to implement people’s way of walking based on radar spectrograms and range-Doppler maps. Wang et al. [28] constructed a range-time map (RTM), Doppler-time map (DTM) and angle-time map (ATM) based on FMCW radar. The k-means algorithm was used to cluster the central time-frequency trajectory of each gesture spectrum, and the Fusion Dynamic Time Warping (FDTW) algorithm was used for gesture classification.

Recently, Transformer [29] has shown great success in the field of natural language processing (NLP) [30,31], computer vision [32,33] and point cloud [34,35]. In this paper, we propose a transformer-based framework for radar gesture recognition, namely RGTNet. It fully extracts the spatial and temporal information of the input radar features to better guide the gesture recognition task. Experimental results show that our proposed method achieves the highest classification accuracy.

Our contributions are summarized as follows.

(1) We collected and made a gesture recognition dataset based on MMW radar in an in-vehicle environment. Based on the FMCW radar, we carried out gesture acquisition under actual driving conditions. By radar signal processing, we converted the original continuous IF signals into time-sequenced RDMs, RAMs, and REMs. Then we preprocessed the radar feature maps through ROI extraction, vibration removal algorithm, background removal algorithm and standardization to obtain cleaner data. Finally, we obtained a total of 5318 samples containing eight types of gestures. Each sample contains eight continuous frames of the processed RDMs, RAMs, and REMs.
(2) We propose a transformer-based network architecture for radar gesture recognition, RGTNet. The spatial feature extraction and spatial feature fusion modules are designed to embed the input of the time-sequenced radar feature maps. We take the temporal information between frames into account by positional encoding. A transformer encoder based on VIT [32] is used to extract the deep temporal feature associations between different frames. Finally, the probability prediction is completed by the fully connected layer and softmax operation.
(3) The experimental results show that our proposed method can better extract radar spatial-temporal features and get a high level of accuracy.

**Figure 1:**
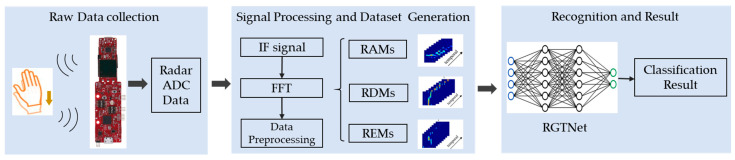
The overview of the proposed gesture recognition system.

The gesture recognition system proposed in this paper mainly contains three parts: data acquisition, signal processing and dataset generation, and gesture recognition algorithm, as shown in Figure 1. We design several typical gestures in the in-vehicle environment for data acquisition. Then, we use radar signal processing and data preprocessing to obtain our dataset, which includes RDMs, RAMs, and REMs of different gestures. In the recognition stage, we train the proposed RGTNet to complete the gesture classification.

The remainder of this paper is organized as follows. Section 2 describes the production of the radar gesture recognition dataset, including data acquisition, radar signal processing, and data preprocessing. Section 3 describes our RGTNet architecture and the components of each module. Section 4 conducts experiments and results analysis. Finally, in Section 5, we give conclusions and some future research.

## 2. Dataset Production

### 2.1. Experimental Equipment and Data Acquisition

**Figure 2:**
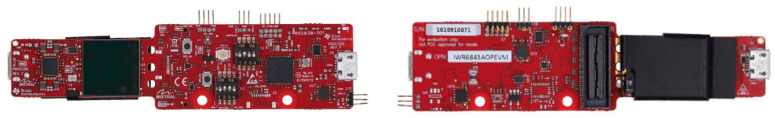
IWR6843AOP appearance diagram.

The IWR6843AOP [36] from Texas Instruments was chosen for the hardware part of the system designed in this paper for data acquisition. The radar is a 60 GHz MMW radar with a bandwidth of 4 GHz, thus facilitating the data acquisition and accurate recognition of subtle motion. With four receivers and three transmitters, the radar can measure the range of multiple objects, Doppler information, and calculate the azimuth and elevation of objects. The appearance diagram and parameter list of the radar are shown in Figure 2 and Table 1, respectively.

**Figure 3:**
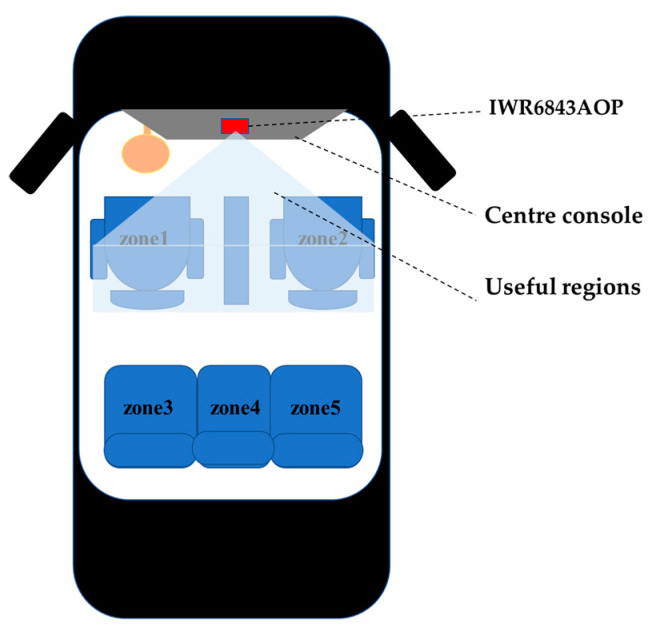
Experiment environment.

We collected raw data in the in-vehicle environment, as shown in Figure 3. The radar was fixed above the centre console. The in-vehicle environment is quite different compared to other environments, such as laboratory environments. First of all, the space inside the vehicle is very narrow. In order to ensure the accuracy and efficiency of the gesture recognition algorithm, we need to select the useful regions in the radar feature map, which is shown in Figure 3. Secondly, variable driving conditions will produce different degrees of vibration, such as acceleration, deceleration and idling, which has a greater impact on the Doppler feature of the radar. However, most environmental settings do not consider vibration. Finally, the closed space inside the vehicle has a more complex background than other environments, including handles, centre boxes, various metal objects and seats of the vehicle. Therefore, for the gesture recognition system under an in-vehicle environment, we need to obtain signals in the useful regions and effectively suppress the effects of background and vibration.

**Figure 4:**
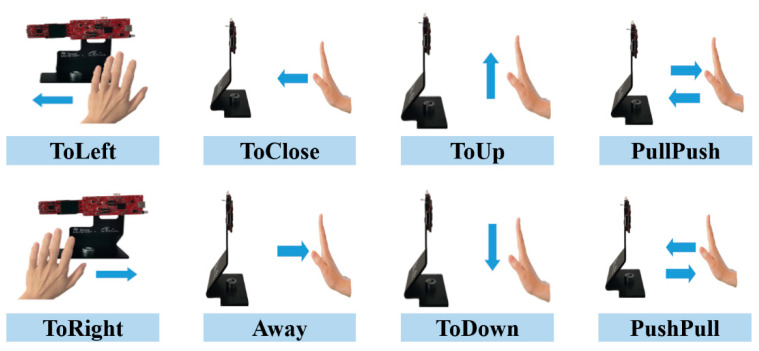
The types of hand gestures.

In this paper, we design eight types of hand gestures: ToLeft, ToRight, ToClose, away, ToUp, ToDown, PullPush, and PushPull, as shown in Figure 4. We had a total of six subjects, each in a group of two, in the driver’s and co-driver’s seats for data collection. During data acquisition, we ensured that each gesture was made complete, and the speed of each gesture was moderate. Each gesture was completed in about 2 s, with the speed range of 0.1 m/s–0.5 m/s and the distance from hand to the radar of between 0.1–0.9 m.

All raw data were read through the serial port. We resolved the RDM, RAM and REM for each frame through radar signal processing, as described in Section 2.2. The data were collected under in-vehicle conditions, and the overall status of the vehicle included static and motion. When the vehicle was in motion, it mainly included acceleration, deceleration, uniform speed or turning conditions. In contrast, the static state mainly included stopping or idling. In the motion status, we acquired continuous data streams and recorded the whole procedure synchronously with the camera to label the gestures. We recorded eight frames of gesture data in a fixed two seconds each time and labelled them in the static case.

To obtain the corresponding gesture sequence from a large amount of data stream under the motion conditions, we need to know the start and end frames of each gesture. We used the Gaussian mixture model [37] and support vector machine [38] to filter out frames with pure background and interfering actions to find the starting frame of each gesture. Then, since each gesture lasted about two seconds, we intercepted eight consecutive frames from the starting frame as the entire gesture sequence. The whole process is similar to the process of intercepting a sequence in a sliding window. This part is not the focus of this paper, so it is not be repeated.

We initially obtained 5318 gesture samples, each containing eight consecutive RDMs, RAMs, and REMs. To minimize the impact of complex background and noise vibration on recognition, we performed subsequent preprocessing on the obtained samples described in Section 2.3.

### 2.2. Radar Signal Processing

The MMW radar signal processing includes two processes: analogue-to-digital conversion and digital signal processing [39]. First, the radar transmits the chirp signal through three transmitting antennas. The transmitted signal is expressed as

(1) $$s_{T}(t)=A_{T}\cos(2\pi f_{c}t+2\pi \int_{0}^{t}f_{T}(\tau )d\tau )$$

where $A_{T}$ represents the transmitted signal amplitude, $f_{c}$ is the carrier frequency, $f_{T}(\tau )=\tau B/T$ is the transmit frequency as a linear function of time, B is the bandwidth, and T is the time duration.

The received signal can be expressed as

(2) $$s_{R}(t)=A_{R}\cos(2\pi f_{c}(t-t_{d})+2\pi \int_{0}^{t}f_{R}(\tau )d\tau )$$

where $A_{R}$ is the received signal amplitude, $t_{d}$ is the delayed time, and $f_{R}(\tau )$ is the frequency of the received signal. By mixing the transmitted and the received signals, we get the intermediate frequency (IF) signal, which is forwarded to the low-pass filter and can be expressed as

(3) $$s_{IF}(t)=\frac{1}{2}\cos(2\pi f_{c}t_{d}+2\pi (\frac{B}{T}t_{d}-f_{d})t)$$

where $f_{d}$ is the Doppler shift caused by the movement of gestures.

**Figure 5:**
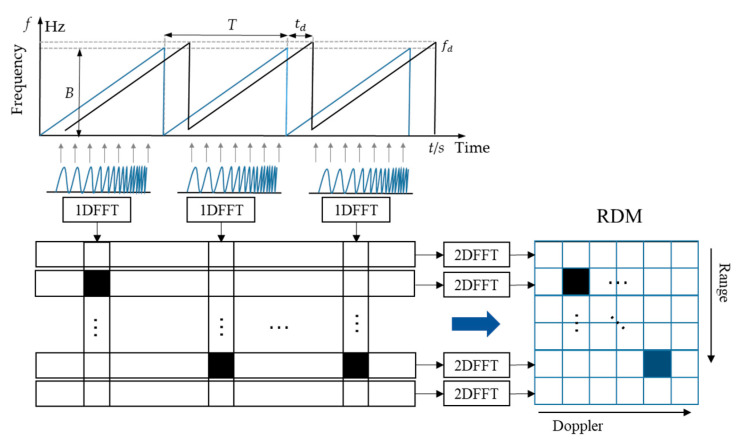
The generation process of RDM.

In signal processing, the IF signal is firstly transformed by the fast time dimension FFT to obtain the one-dimensional range-Doppler sequence. The sequences contain the position information of the gestures, which can be represented by the peak of the sequences. Then the RDM is obtained by extracting the components of the same frequency of each pulse by using the 2D FFT, as shown in Figure 5.

**Figure 6:**
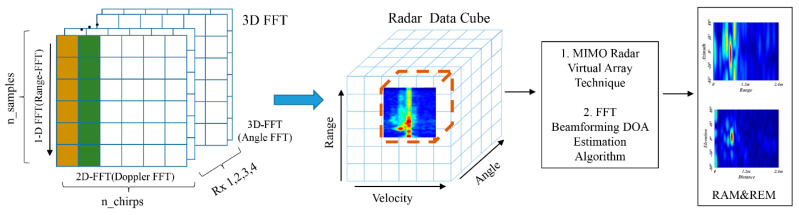
The generation process of RAM and REM.

The gesture trajectory has different azimuth and elevation angles, which can be estimated by adding an angle FFT, as shown in Figure 6. Based on the RDM obtained by two-dimension FFT, the Radar Data Cube consisting of Range-Doppler-Angle can be obtained by adding the angle FFT transformation of the antenna dimension. We select a specific static velocity slice to obtain the instantaneous static range angle map. The multiple-input multiple-output (MIMO) virtual array technique and the FFT beamforming direction of arrival (DOA) estimation algorithm [40] are used to get a higher resolution range-angle map. For the RAM, the information of the horizontal antenna can be selected for angle FFT; For the REM, the information of the vertical antenna can be selected for angle FFT.

### 2.3. Data Preprocessing

We further process the radar feature maps obtained by radar signal processing, including the ROI extraction, vibration removal algorithm, background removal algorithm, and standardization. Using the related algorithms, we get pure input data, thus ensuring the accuracy and generalization of the recognition algorithm.

#### 2.3.1. The ROI Extraction

**Figure 7:**
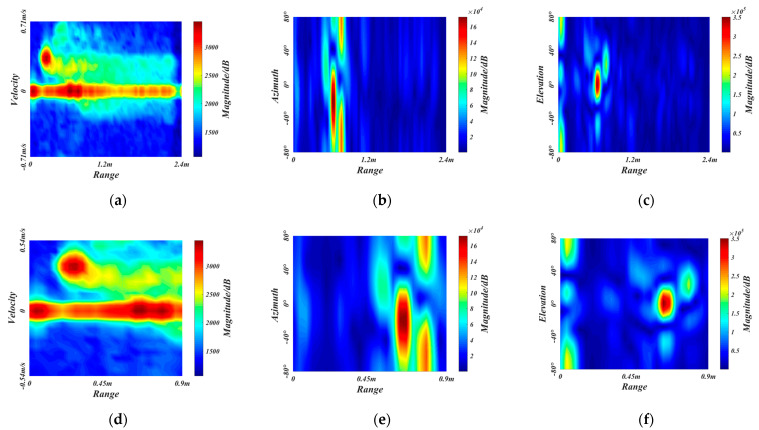
The ROI extraction. (**a**–**c**) are the original RDM, RAM, and REM, respectively. (**d**–**f**) are the ROI extraction results of RDM, RAM, and REM, respectively.

To reduce the difficulty of network training and prevent interference from the rear passengers and invalid regions, we crop off the redundant sizes of radar feature maps to obtain the ROI. The gesture recognition considered in this paper is applied in the vehicle, where the primary users are the driver and the co-driver. Therefore, the maximum recognition range of the radar is selected as 0.9 m, which can cover the front areas. Since the gesture speed is moderate and the whole gesture takes about 2 s, the maximum radar recognition speed is selected as 0.54 m/s. As for the detection field angle of radar, we empirically choose the horizontal angle and elevation angle to be −48°~48°. Figure 7a–c are the original RDM, RAM, and REM of a frame belonging to close gesture, respectively. The ROI extraction results of RDM, RAM, and REM are shown in Figure 7d–f, respectively.

#### 2.3.2. Vibration Removal Algorithm

Due to the mechanical parts jittering and road inequality, the vehicle will produce a certain degree of vibration under driving or idling conditions. The vibration can affect the intensity distribution of the range-Doppler map. In the range-Doppler map, the vibration is mainly concentrated in the interval of −0.2–0.2 m/s, which mainly shows the abnormal high-intensity regions caused by vibration. We should remove these vibrational noises and micro-motions of static objects to prevent them from interfering with gesture features.

We define I(r,v,t) as the intensity value at range r and velocity v in the range-Doppler map at the time t. The μ(r,v,t) and σ(r,v,t) are the defined mean and standard deviation at the time t, respectively, where v∈[−0.2,0.2] and r∈[0,0.9]. The updating formula is expressed as

(4) $$\left\{ \begin{array}{l} \mu (r,v,t)=(1-\alpha )\mu (r,v,t-1)+\alpha I(r,v,t)\\ \sigma^{2}(r,v,t)=(1-\alpha )\sigma^{2}(r,v,t-1)+\alpha {\left[I(r,v,t)-\mu (r,v,t-1)\right]}^{2} \end{array}\right.$$

where α is the updating rate, which is related to the intensity variation rate $\delta_{t}$, which can be expressed as

(5) $$\delta_{t}=\frac{I(r,v,t)-\mu (r,v,t-1)}{\mu (r,v,t-1)}$$

The updating rate α should increase with the intensity variation rate $\delta_{t}$ to remove the localized high-intensity regions caused by vibration, which is modelled as

(6) $$\alpha =\left\{ \begin{array}{l} \frac{e^{\delta_{t}}-e^{-\delta_{t}}}{e^{\delta_{t}}+e^{-\delta_{t}}},\delta_{t}\geq 0\\ 0.5\times (\frac{e^{-\delta_{t}-2}-e^{\delta_{t}+2}}{e^{\delta_{t}+2}+e^{-\delta_{t}-2}}+1),\delta_{t}<0 \end{array}\right.$$

**Figure 8:**
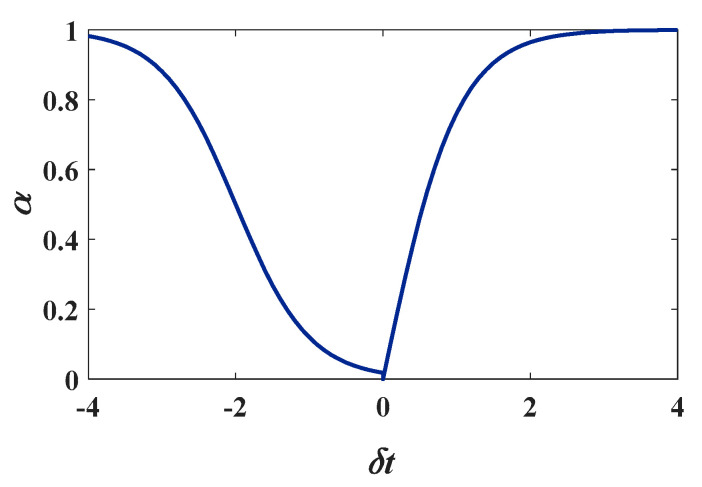
The $\alpha -\delta_{t}$ curve.

Figure 8 shows the relationship curve of α with $\delta_{t}$. The updating rate α increases smoothly when the intensity variation rate $\delta_{t}$ increases, making the mean μ close to the high-intensity vibration.

We set the mask matrix to be M, whose size is equal to the range-Doppler map, i.e., 24×24. The value of M is 1 when |v|>0.2, and the other values satisfy the following equation.

(7) $$M(r,v,t)=\left\{ \begin{array}{l} 0,\left|I(r,v,t)-\mu (r,v,t)\right|\leq \sigma (r,v,t)\\ 1,\left|I(r,v,t)-\mu (r,v,t)\right|>\sigma (r,v,t) \end{array}\right.$$

with the mask matrix M, we can get the range-Doppler map after removing the vibration, which is represented as $I_{O}$.

(8) $$I_{o}(r,v,t)=M(r,v,t)\cdot I(r,v,t)$$

**Figure 9:**
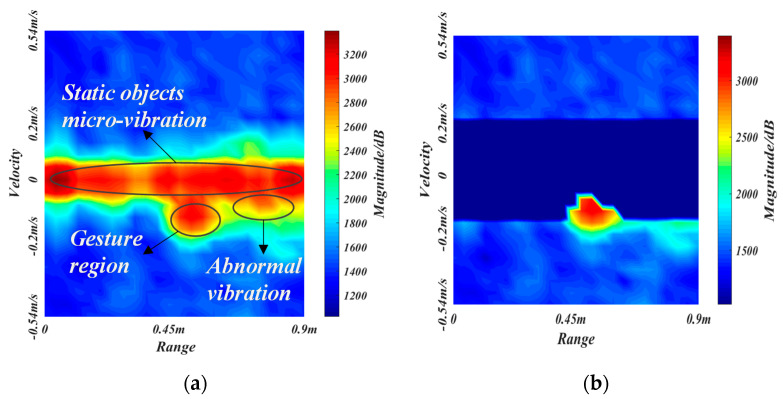
The results of the vibration removal algorithm. (**a**,**b**) are the figure before and after vibration removal, respectively.

Figure 9a,b shows the RDM before and after removing the vibration. Our algorithm can retain the gesture region well and filter out the micro-vibration objects and local anomaly regions for subsequent processing.

#### 2.3.3. Background Removal Algorithm

To remove the interference of cluttered background on gesture features, we use the frame-difference method of dynamic thresholding to remove the background. We use a unidirectional queue to save the sixteen background frames nearest the current time. Set each background frame as $B_{1}$, $B_{2}$, …, $B_{16}$. $\bar{B}$ is the mean value of the background, and its dynamic update formula is as follows

(9) $$\bar{B}=\frac{16\times {\bar{B}}^{\prime }-B_{16}+B_{new}}{16}$$

where $B_{new}$ is the newly added background frame, ${\bar{B}}^{\prime }$ is the mean value before the new background frame is added. $B_{new}$ is added to the end of the queue and $B_{16}$ is removed from the head of the queue. The updating formula for the background queue is as follows

(10) $$B_{i}=\left\{ \begin{array}{l} B_{i-1},i=2,3,\ldots ,16\\ B_{new},i=1 \end{array}\right.$$

We set ΔI to be the frame difference between the mean of the system background and the gesture frame I at the time t. It can be expressed as

(11) $$\Delta I=I-\bar{B}$$

We construct the mask matrix $I_{M}$ with the same size as the input feature map, which can be described as

(12) $$I_{M}(x,y,t)=\left\{ \begin{array}{l} 0,\Delta I(x,y,t)<T\\ 1,\Delta I(x,y,t)\geq T \end{array}\right.$$

where T is the threshold. The feature map after removing the background is $I_{P}$, which is calculated as

(13) $$I_{P}(x,y,t)=I_{M}(x,y,t)\cdot I(x,y,t)$$

Since our experiment contains different subjects with different gestures and driving conditions, it is unsuitable to take a fixed threshold T. Through the analysis of many gesture frames, we find that most background regions unrelated to gestures can be filtered out when the frame difference is retained in the top 10% of the feature map. It ensures the feature completeness of the gesture regions. Therefore, the dynamic threshold is set as the minimum value of the top 10% of ΔI.

**Figure 10:**
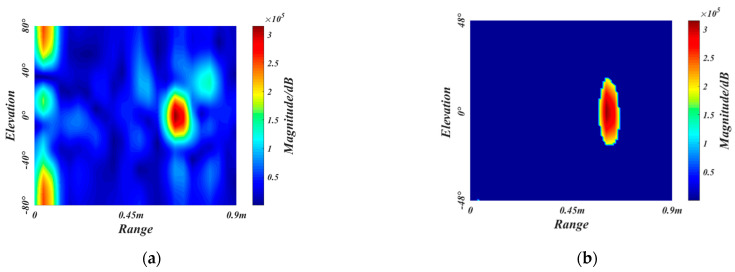
The results of the background removal algorithm. (**a**,**b**) are the figure before and after background removal, respectively.

We use the minimum heap to compute the threshold dynamically to reduce the time complexity and space complexity. Figure 10a,b shows a REM before and after removing the background.

#### 2.3.4. Standardization

To facilitate the training of the network, we normalized the feature maps. Set the current feature map as I, and the width and height of the feature map as w and h, respectively. Then the mean μ and variance $\sigma^{2}$ are expressed as

(14) $$\left\{ \begin{array}{l} \mu =\frac{\sum_{i=1}^{w}\sum_{j=1}^{h}I(x,y)}{w\times h}\\ \sigma^{2}=\frac{\sum_{i=1}^{w}\sum_{j=1}^{h}{\left(I(x,y)-\mu \right)}^{2}}{w\times h} \end{array}\right.$$

The standardized feature map is $I_{new}$, which is expressed as

(15) $$I_{new}(x,y)=\frac{I(x,y)-\mu }{\sigma }$$

**Figure 11:**
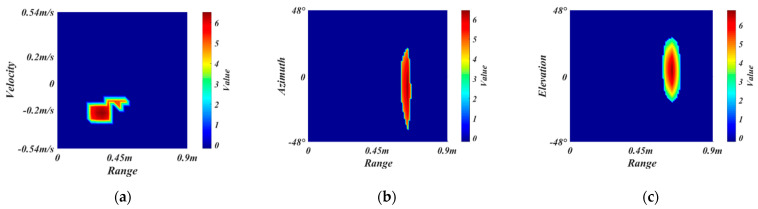
Standardization. (**a**–**c**) are the standardized RDM, RAM, and REM, respectively.

Figure 11a–c shows the final RDM, RAM, REM of a frame, respectively.

## 3. Proposed Method

Our network is based on the transformer, which we named RGTNet. The input of the network consists of three branches, i.e., RDMs, RAMs and REMs. The network can sufficiently extract the spatial-temporal features of the input data and finally output the gesture prediction probability.

### 3.1. Network Architecture

**Figure 12:**
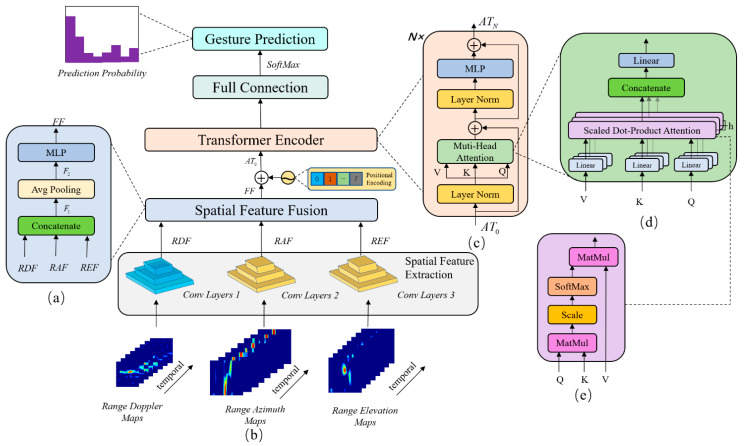
The architecture of RGTNet. (**a**) is the structure of spatial feature fusion module. (**b**) is the overall architecture of RGTNet. (**c**) is the structure of Transformer encoder. (**d**) is the structure of multi-head attention module. (**e**) is the calculation flow of scaled dot-product attention.

The overall architecture of RGTNet is shown in Figure 12. There are three branches of network input, which are RDMs, RAMs, and REMs. Each input branch consists of eight consecutive frames stacked in a temporal sequence. The network first extracts the spatial features through the convolution Layers1, convolution Layers2 and convolution Layers3 in Figure 12b and stacks them in time sequence to obtain the range-Doppler features (RDF), range-azimuth features (RAF), and range-elevation features (REF). Then the three features are fed into the spatial feature fusion module for feature fusion between different modalities. The output FF is the fused spatial features stacked in a time sequence.

Inspired by transformer and VIT, we adapt the transformer structure with attention mechanism as the core to further extract inter-frame information in the temporal dimension. We regard FF as an embedded feature. Since there is temporal information between frames, we add the position encoding and get $AT_{0}$. We stack N transformer encoder modules to extract the deep features between different frames fully. Finally, we use softmax operation to process the features after full connection and output the predicted probability of each gesture. The gesture corresponding to the maximum probability is the classification result of the network.

### 3.2. Spatial Feature Extraction

**Figure 13:**
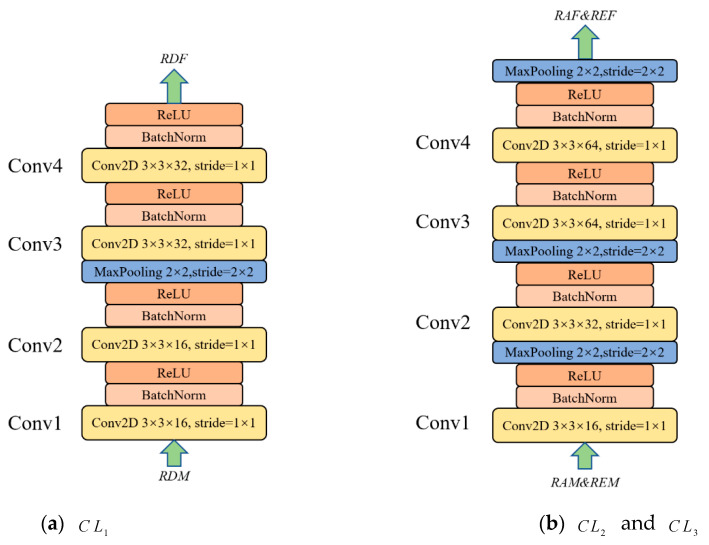
The structure of the spatial feature extraction module.

The inputs of the spatial feature extraction module are RDMs, RAMs and REMs, where $RDMs\in {\mathbb{R}}^{t\times c_{D}\times h_{D}\times w_{D}}$, $RAMs\in {\mathbb{R}}^{t\times c_{A}\times h_{A}\times w_{A}}$, $REMs\in {\mathbb{R}}^{t\times c_{E}\times h_{E}\times w_{E}}$. Each modality is consecutive t frames. For RDMs, each frame has a size of $h_{D}\times w_{D}$ with $c_{D}$ channels. The RAMs and REMs are similar. We use three independent convolution layers for spatial feature extraction with a structure similar to VGGNet [41], i.e., a 3 × 3 convolution and pooling layer with batch normalization and ReLU activation function. The Conv Layers2 and Conv Layers3 use the same structure since the RAMs and REMs are of the same size. However, their parameters are not shared. Figure 13 shows the structure of Conv Layers1 ($CL_{1}$), Conv Layers2 ($CL_{2}$) and Conv Layers3 ($CL_{3}$).

Formally, we express the whole process as

(16) $$\left\{ \begin{array}{l} RDF=CL_{1}(RDM^{1})⊕CL_{1}(RDM^{2})⊕\cdot \cdot \cdot ⊕CL_{1}(RDM^{t})\\ RAF=CL_{2}(RAM^{1})⊕CL_{2}(RAM^{2})⊕\cdot \cdot \cdot ⊕CL_{2}(RAM^{t})\\ REF=CL_{3}(REM^{1})⊕CL_{3}(REM^{2})⊕\cdot \cdot \cdot ⊕CL_{3}(REM^{t}) \end{array}\right.$$

where $RDM^{i}$, $RAM^{i}$, $REM^{i}$ denotes the *i*-th (i∈{1,2,…,t}) frame in RDMs, RAMs and REMs. The ⊕ is the concatenation operation, which is to stack the extracted features of each frame in a temporal sequence. In this way, we obtain the spatial features, i.e., $RDF\in {\mathbb{R}}^{t\times \tilde{c_{D}}\times \tilde{h_{D}}\times \tilde{w_{D}}}$, $RAF\in {\mathbb{R}}^{t\times \tilde{c_{A}}\times \tilde{h_{A}}\times \tilde{w_{A}}}$ and $REF\in {\mathbb{R}}^{t\times \tilde{c_{E}}\times \tilde{h_{E}}\times \tilde{w_{E}}}$. In this paper, the final number of extracted feature channels is 32, 64, 64, and the feature map size is 12 × 12.

### 3.3. Spatial Feature Fusion

The spatial feature fusion module is shown in Figure 12a. In this module, we fuse the spatial features of different modalities to obtain the feature representation of each frame for further temporal analysis. Firstly, we concatenate the features of different modalities in the channel dimension, i.e., Equation (5)

(17) $$F_{1}=RDF⊕RAF⊕REF$$

where $F_{1}\in {\mathbb{R}}^{t\times (\tilde{c_{D}}+\tilde{c_{A}}+\tilde{c_{E}})\times h\times w}$, and the size of the feature map h×w is 12 × 12 mentioned above. Then, after averaging pooling and multi-layer perception machine, we obtain the fused feature FF and can be expressed as

(18) $$FF=MLP(AvgPooling(F_{1}))$$

where AvgPooling(⋅) indicates the average pooling at each feature map. The MLP(⋅) consists of two linear layers and a ReLU activation function for the final spatial feature integration. $FF\in {\mathbb{R}}^{t\times l}$ is the final fusion feature.

### 3.4. Transformer Encoder Module

The transformer has achieved great success in the NLP and CV fields. Inspired by it, we use the transformer encoder to capture the feature association between different frames, as shown in Figure 12c. In this paper, we regard the spatial fusion feature FF as the feature after input embedding in transformer. Due to the strict sequential relationship between different frames, just as with the sequential relationship between different words in NLP, we need to encode the position of the input sequence feature. We use position encoding in transformer, which is expressed as

(19) $$\left\{ \begin{array}{l} PE(pos,2i)=\sin(pos/1000^{2i/d_{m}})\\ PE(pos,2i+1)=\cos(pos/10000^{2i/d_{m}}) \end{array}\right.$$

where PE is the position encoding, pos represents the position while i represents the corresponding dimension. The $d_{m}$ is the output dimensions of each transformer encoder, which is also equal to the embedded feature dimension l. Our transformer encoder structure is as described in VIT. Nevertheless, we use spatial fusion information as the input embedding, and there is no additional learnable position encoding. The whole process is expressed as

(20) $$\left\{ \begin{array}{l} AT_{0}=FF+PE,FF\in {\mathbb{R}}^{t\times l},PE\in {\mathbb{R}}^{t\times l}\;\\ \bar{AT_{m}}=MHA(LN(AT_{m-1}))+AT_{m-1},m=1,2,\ldots ,N\\ AT_{m}=MLP(LN(\bar{AT_{m}}))+\bar{AT_{m}},m=1,2,\ldots ,N \end{array}\right.$$

where $\bar{AT_{m}}\in {\mathbb{R}}^{t\times d_{m}}$ is the intermediate result of the *m*-th transformer encoder, and $AT_{m}\in {\mathbb{R}}^{t\times d_{m}}$ is the output result of the *m*-th transformer encoder. The LN(⋅) represents layer normalization and MHA(⋅) represents multi-head attention operation. The MLP(⋅) consists of two linear layers and a GeLU activation function.

For the multi-head attention module, its structure is shown in Figure 12d. It enables the model to learn and represent features in different subspaces. The core operation of it is Scaled Dot-Product Attention (SA), as shown in Figure 12e. This operation can be expressed as

(21) $$\left\{ \begin{array}{l} MHA(Q,K,V)=(head_{1}⊕head_{2}⊕\ldots ⊕head_{h})W^{O}\;\\ head_{i}=SA_{i}(QW_{i}{}^{Q},KW^{K}{}_{i},VW^{V}{}_{i}),i=1,2,\ldots ,h\\ SA_{i}(QW_{i}{}^{Q},KW^{K}{}_{i},VW^{V}{}_{i})=softmax(QW_{i}{}^{Q}{\left(KW_{i}{}^{K}\right)}^{T}/\sqrt{d_{k}})VW_{i}^{V},i=1,2,\ldots ,h \end{array}\right.$$

where Q,K,V correspond to the output of the first layer norm in the Transformer encoder, the linear projection matrices are $W_{i}^{Q}\in {\mathbb{R}}^{d_{m}\times d_{k}}$, $W_{i}^{K}\in {\mathbb{R}}^{d_{m}\times d_{k}}$, $W_{i}^{V}\in {\mathbb{R}}^{d_{m}\times d_{v}}$, and $W^{o}\in {\mathbb{R}}^{hd_{v}\times d_{m}}$. We follow the transformer and add the scaling factor $1/\sqrt{d_{k}}$ to the dot product. In this paper, we take the number of heads as eight and $d_{k}=d_{v}=d_{m}/h=16$.

### 3.5. Full Connection Outhead

We use the max pooling for $AT_{N}$ obtained by the transformer encoder and feed the obtained features into the fully connected layer for final classification. Our fully connected classifier consists of two linear layers and a ReLU activation function. The number of hidden units is 64. The softmax operation achieves the final probability prediction where the gesture class corresponding to the probability maximum is the prediction result of the network.

## 4. Experiment

### 4.1. Experimental Details and Evaluation Metrics

Our dataset contains a total of 5318 gesture samples, each of which contains eight consecutive RDMs, RAMs, and REMs. We have performed the preprocessing according to the method in Section 2.3. We randomly divide the data into a training set and a test set in the ratio of 7:3.

Our implementation of RGTNet is based on PyTorch [42]. We use the Adam optimizer [43] for end-to-end network training. Since gesture recognition is a multi-classification task, we choose the cross-entropy loss function. Our batch size is set to 8, the initial learning rate is 0.001, and the weight decay is 0.0001. We train a total of 150 epochs, and all experiments are conducted with a GeForce GTX 1080ti.

For multi-gesture classification tasks, overall precision, single-class precision and confusion matrix are commonly used quantitative evaluation metrics. The overall accuracy is the number of correctly predicted samples in the test set as a percentage of the total number of samples in the test set. The single-class accuracy is the number of correctly predicted samples for each class of gestures in the test set as a percentage of the total number of samples for that class. It measures the classification effectiveness of the model for each class. On the other hand, the confusion matrix counts the classes of correct and incorrect gesture predictions for each gesture. It aggregates all of them into a table that is used to represent the confusion between different classes. It gives a visual representation of the specific performance of the model.

### 4.2. Experimental Results

To fully validate the effectiveness of our proposed algorithm, we compare different advanced methods used in gesture recognition. To adapt the input features and sizes of our dataset, we make some adaptations to the original network. In the 2DCNN-based method [19], we preserve the three-branch 2DCNN of our method and use the spatial fusion features FF as the final extracted features. In the CNN-LSTM-based method, we feed the spatial fusion features FF into the LSTM for temporal analysis. In the 3DCNN-based method, we use a similar structure to C3D [44] with three branches for spatial-temporal feature extraction. The rest of the training parameters are kept consistent. The overall accuracy (OA) and single-class accuracy of the test set are shown in Table 2.

It can be seen from the test results that the algorithm we proposed achieves the best recognition accuracy of 97.56%. At the same time, the methods based on 2DCNN, CNN-LSTM and 3DCNN are 92.50%, 95.76%, and 95.21%, respectively. For single-class classification accuracy, our algorithm achieves optimal results on Toleft, ToClose, ToUp, PullPush, and PushPull gestures, which fully demonstrates the effectiveness of the model. For the 2DCNN-based method, the accuracy is low since it only extracts spatial information and does not fully extract temporal features. Both CNN-LSTM-based and 3DCNN-based methods can extract spatial-temporal features. However, in comparison, our Transformer-based structure can better learn and represent the intrinsic association between frames and better describe the features.

**Figure 14:**
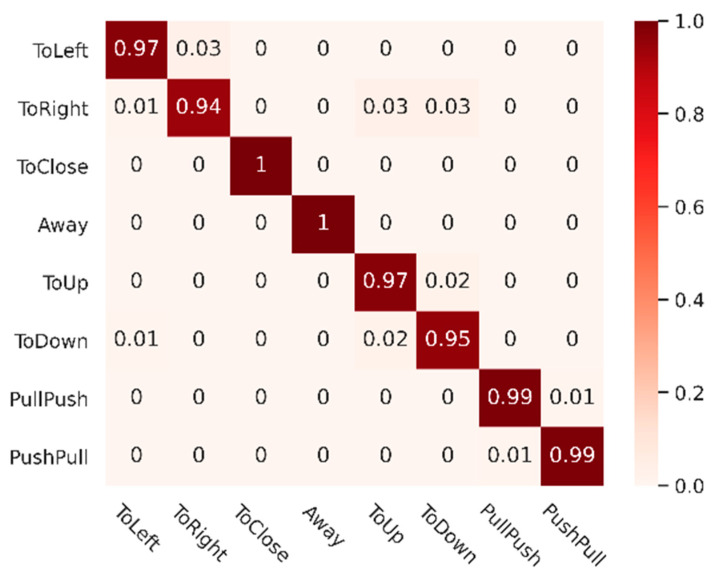
Confusion matrix of RGTNet with our dataset.

To illustrate the degree of confusion between different classes of our method, we plot the confusion matrix as shown in Figure 14 and keep two decimal places for the results. As we can see, the test results of our method are promising in most cases, but there are still some confusing errors. The ToRight gesture is easily confused with ToUp and ToDown, as well as between ToUp and ToDown. Intuitively, these gestures have certain similarities. For example, when making a ToRight gesture in the vehicle, if the gesture is not kept perfectly horizontal by vibration interference, there may be a tendency to down.

## 5. Discussion

### 5.1. Impact of Different Input Modalities

**Figure 15:**
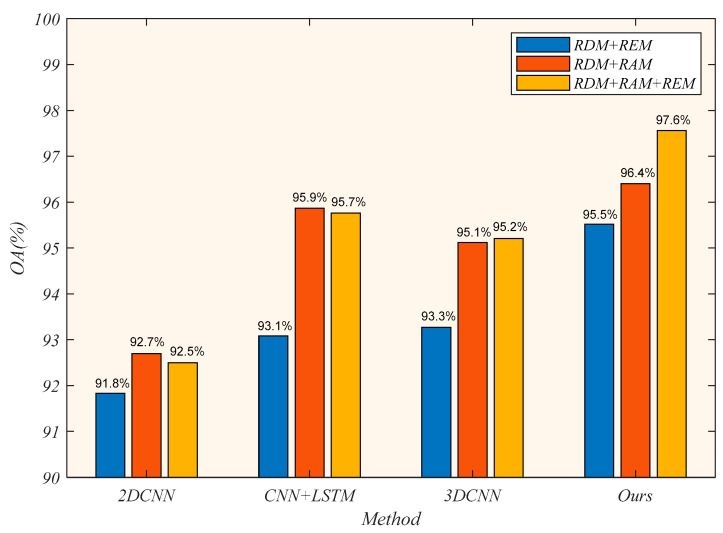
The overall accuracy using different combinations of modalities.

To explore the effect of different input modalities on the classification results, we compare the test results of each model under three different input combinations, shown in Figure 15. The input combinations are RDM+REM, RDM+RAM, RDM+RAM+REM, respectively.

We adjust the number of convolutional kernels to adapt to different input modalities, ensuring the consistency of the extracted feature dimensions. As we can see, our method achieves optimal results for all three different inputs, with overall accuracies of 95.5%, 96.4% and 97.6%, respectively. In general, when the input modality is RDM+REM, the recognition accuracy of each method is low. It indicates that the Doppler information and the elevation information do not adequately represent the gesture features, resulting in worse classification results. Furthermore, when the input modalities are RDM+RAM and RDM+REM+RAM, the 2DCNN-based method, CNN-LSTM-based method and 3DCNN-based method achieve approximate results. These three methods can make full use of the RDM and RAM information and are sufficient to see the importance of the two inputs information. However, when the input modality of our method is RDM+RAM+REM, the accuracy is still 1.2% higher than that of the input RDM+RAM. Therefore, the transformer-based structure can use the information of the three input features and obtain the internal correlation between frames to get the best classification results.

### 5.2. Impact of Numbers of Transformer Encoder

**Figure 16:**
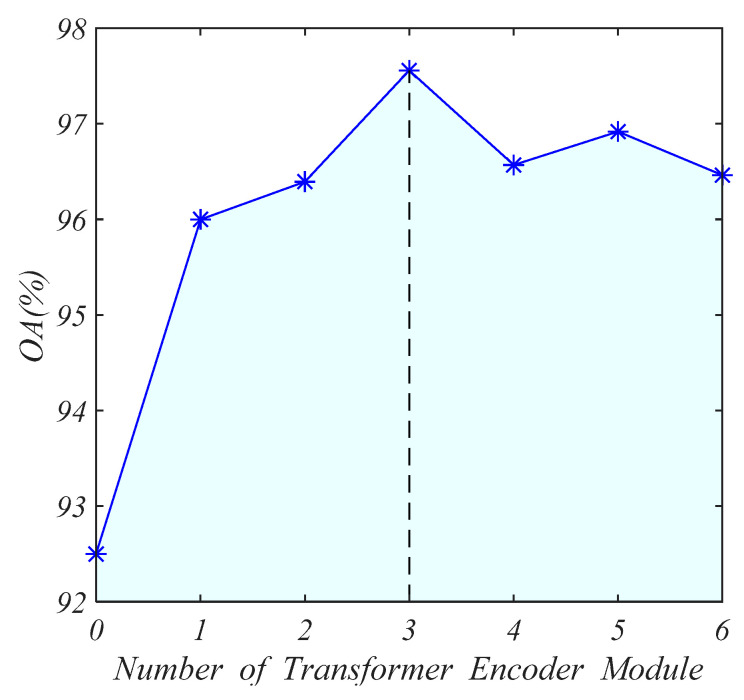
The influence of the number of transformer encoder modules.

In our network, the number of transformer encoders N is an important parameter. More encoders mean deeper feature extraction, but it brings more parameters and makes the model more complex. We set the number of transformer encoders from 0 to 6. The accuracy of the test set is shown in Figure 16. When there is no transformer encoder module (i.e., only 2DCNN is used to extract features), the accuracy of the test set is low. As the number of encoders increases, the accuracy of the test set gradually increases. When N is 3, the accuracy is the highest, and then the accuracy does not increase. Therefore, the prediction accuracy and complexity of the model reach a good balance when N is 3.

### 5.3. Impact of the Size of the Train Set

**Figure 17:**
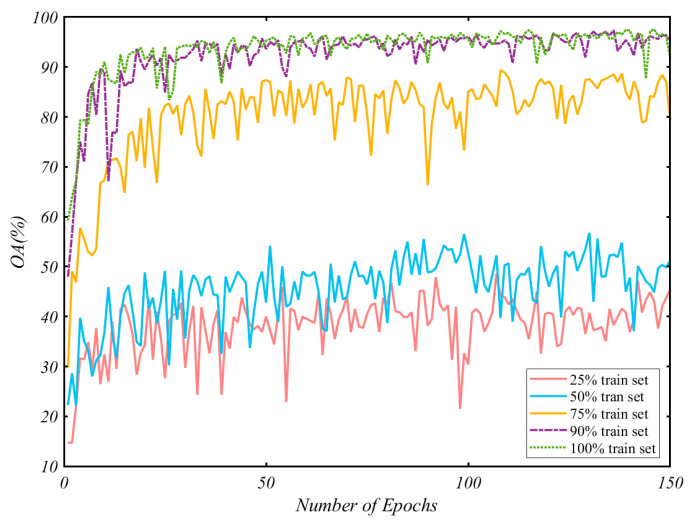
The overall accuracy varying with the number of epochs.

To fully validate the effectiveness of the proposed method, we analyze the performance of the proposed method under the different sizes of the train set. We train the network using 25%, 50%, 75%, and 90% of the train set and compare the results with the previous training using the entire dataset. The overall accuracy curve of the test set with the training epoch is shown in Figure 17. We can see that the accuracy of the model quickly reaches the bottleneck on the small training set, and the results are not very stable, with more significant oscillations. With the increase of training data, the accuracy of the test set increases. When the training set is 90%, the network performance is generally consistent with the results obtained from the entire data set. It indicates that our network has good expressiveness and generalization performance.

## 6. Conclusions

In this paper, we proposed a gesture recognition algorithm, namely RGTNet, which is based on FMCW radar and Transformer. Firstly, we collected eight typical dynamic gesture data in an in-vehicle environment. Then we obtained a dataset containing continuous RDMs, RAMs and REMs by radar signal processing and data preprocessing. Finally, we propose RGTNet, which contains a spatial feature extraction module, spatial feature fusion module and transformer encoder modules. The RGTNet can fully present the spatial-temporal information of the radar feature maps to accomplishing gesture recognition better. Experimental results show that our algorithm achieves better results than the mainstream methods, with an overall accuracy of 97.56%. We will investigate better models to improve the accuracy further and consider more relevant in-vehicle applications in future research.

## Figures and Tables

**Table 1:** The key parameters of the radar.

Parameters	Value
Start Frequency	60 GHz
Bandwidth	4 GHz
Frequency Slope	100 MHz/μs
Chirps per frame	96
Maximum range	2.4 m
Range resolution	0.0469 m
Maximum angle	−80°–80°
Angle resolution	29°
Maximum velocity	0.7120 m/s
Velocity resolution	0.0445 m/s
Antennas	3 × TX, 4 × RX

**Table 2:** Gesture classification results.

Method	OA	ToLeft	ToRight	ToClose	Away	ToUp	ToDown	PullPush	PushPull
2DCNN-based	92.50%	92.34%	84.72%	92.99%	100%	97.13%	98.14%	87.04%	87.96%
CNN+LSTM	95.74%	92.79%	94.44%	93.46%	99.47%	95.69%	94.42%	95.83%	99.44%
3DCNN-based	95.21%	93.69%	95.37%	89.72%	98.93%	96.17%	94.42%	95.83%	97.69%
Ours	97.56%	96.85%	93.52%	99.57%	99.53%	97.13%	95.35%	99.07%	99.44%

## Data Availability

The data presented in this study are available on request from the corresponding author.
